# University of the Pacific—Medical Department

**Published:** 1860-05

**Authors:** 


### *University of the Pacific—Medical Department.*

The second session of the Medical Department of the University of the Pacific will commence on the first Monday in May next, and continue eighteen weeks, under flattering auspices.

Good taste does not permit us to speak of the talents, industry or capacity for teaching, of the Faculty of this College, but we will say this, that there is no better place on the globe than San Francisco for establishing a permanent medical school of the first class; and that if the members of the present Faculty should not make it one of the kind, the fault will be their own, because all the materials necessary for accomplishing this object are either here now, or rapidly forming; and will only require to be skilfully appropriated, to succeed. Croakers may assert the contrary notwithstanding.

San Francisco is the finest place in the world for cultivating practical anatomy. It is the only place in which dissections can be conducted the whole year—in July and January alike.

San Francisco is probably the only city in which the climate is *just* right every day in the year for the performance of surgical (plastic excepted) operations, and the advantages of this the statistics of their results can fully show.

These facts being generally known, will, in a few years, bring surgical patients, not only from all the Central American States and the adjacent islands, but in the more remote periods of the future will cause them to come from the Atlantic States, if not from Europe; and wherever an immense number of surgical operations occur, students will always congregate. We are aware that there are persons here who will sneer at this statement. But those who sleep half their time and sneer at what others say and do the balance, are always bad calculators.

Of the immense numbers of young lads now at our literary colleges and schools on this coast there will, in a few years, be many desirous of becoming medical men, and the diseases among us present so many pecular features that in order to practice successfully in this region they must receive a medical education here.

By-the-by, we learn that some of those who sneered most industriously at the idea of a Medical College in California at first, are now talking of establishing a second one in this city. We hope they will; we always did like competition; it affords the finest stimulus to exertion in the world.

Besides, no one can make a respectable teacher in a Medical College without being a hard-worker, and the more active laborers we have in the field of medical science on this coast, the more the profession will be elevated. We feel as if we could become the very friends of those who would perform the labor, and make all the sacrifices necessary for sustaining another Medical College in this city, in spite of conflicting interests which might occur.—*San Francisco Med. Press.*

